# Causal associations between gut microbiota and premature rupture of membranes: a two-sample Mendelian randomization study

**DOI:** 10.3389/fimmu.2024.1440232

**Published:** 2024-09-02

**Authors:** Lei Zhang, Qian Li, Jiafeng Huang, Qin Zou, Hua Zou, Xinyuan Zhang, Yan Su, Chunli Li

**Affiliations:** ^1^ Department of Clinical Laboratory, Chongqing Health Center for Women and Children, Chongqing, China; ^2^ Department of Clinical Laboratory, Women and Children’s Hospital of Chongqing Medical University, Chongqing, China; ^3^ Institute of Pathology and Southwest Cancer Center, Southwest Hospital, Third Military Medical University (Amy Medical University), and The Key Laboratory of Tumor Immunopathology, The Ministry of Education of China, Chongqing, China

**Keywords:** gut microbiota, premature rupture of membranes, genetic variable, Mendelian randomization, causality

## Abstract

**Background:**

Previous study has indicated a potential link between gut microbiota and maternal pregnancy outcomes. However, the causal relationship between gut microbiota and premature rupture of membranes (PROM) remains a topic of ongoing debate.

**Methods:**

A two-sample Mendelian Randomization (MR) study was used to investigate the relationship between gut microbiota and PROM. Genetic data on gut microbiota was obtained from the MiBioGen consortium’s largest genome-wide association study (GWAS) (n=14,306). Genetic data on PROM (3011 cases and 104247 controls) were sourced from publicly available GWAS data from the Finnish National Biobank FinnGen consortium. Various methods including Inverse variance weighted (IVW), MR-Egger, simple mode, weighted median, and weighted mode were utilized to assess the causal relationship by calculating the odd ratio (OR) value and confidence interval (CI). Sensitivity analyses for quality control were performed using MR-Egger intercept tests, Cochran’s Q tests, and leave-one-out analyses.

**Results:**

The IVW method revealed that *class Mollicutes* (IVW, OR=0.773, 95%CI: 0.61-0.981, *pval* = 0.034), *genus Marvinbryantia* (IVW, OR=00.736, 95%CI: 0.555-0.977, *pval* = 0.034), *genus Ruminooccaceae UCG003* (IVW, OR=0.734, 95%CI: 0.568-0.947, *pval* = 0.017) and *phylum Tenericutes* (IVW, OR=0.773, 95%CI: 0.566-1.067, *pval* = 0.034) were associated with a reduced risk of PROM, while *genus Collinsella* (IVW, OR=1.444, 95%CI: 1.028-2.026, *pval* = 0.034), *genus Intestinibacter* (IVW, OR=1.304, 95%CI: 1.047-1.623, *pval* = 0.018) and *genus Turicibacter* (IVW, OR=1.282, 95%CI: 1.02-1.611, *pval* = 0.033) increased the risk of PROM. Based on the other four supplementary methods, six gut microbiota may have a potential effect on PROM. Due to the presence of pleiotropy (*pval*=0.045), *genus Lachnoclostridium* should be ruled out. No evidence of horizontal pleiotropy or heterogeneity was found in other microbiota (*pval >*0.05).

**Conclusions:**

In this study, we have discovered a causal relationship between the presence of specific probiotics and pathogens in the host and the risk of PROM. The identification of specific gut microbiota associated with PROM through MR studies offers a novel approach to diagnosing and treating this condition, thereby providing a new strategy for clinically preventing PROM.

## Introduction

Premature rupture of membranes (PROM) is a prevalent perinatal complication, with an incidence rate of approximately 7% to 8% (1, 2). PROM can result in severe fetal complications such as placental abruption, umbilical cord compression, respiratory distress syndrome, preterm birth, and cerebral damage (3–5). Mothers with PROM face increased risks of intra-amniotic infections, placental abruption, cord prolapse, sepsis, and even death, posing significant threats to both maternal and neonatal health (6, 7). While factors such as infection, inflammation, immunity, oxidative stress, and nutrient metabolism are implicated in the pathogenesis of PROM, reliable early diagnostic indicators and effective preventive measures remain lacking (8, 9).

Gut microbiota, a complex community within the digestive tract, plays a crucial role in nutrient digestion and absorption during energy metabolism. It also maintains physiological functions and regulates various pathological processes in the body (10–12). Numerous studies suggest that gut microbiota plays a significant role in maternal and fetal health, undergoing changes during pregnancy, disruption of maternal gut microbiota during gestation can alter offspring microbiota and immunity (13–16). For example, *Bacteroides fragilis* (*B. fragilis*) dominates the gut microbiomes of individuals with intrahepatic cholestasis of pregnancy (ICP). Through its bile salt hydrolase (BSH) activity, B. fragilis aggravates ICP by inhibiting FXR signaling, thereby disrupting bile acid metabolism (17). In overweight and obese pregnant women at 16 weeks gestation, the abundance of butyrate-producing bacteria and butyrate production in the gut microbiota are significantly negatively associated with blood pressure and with plasminogen activator inhibitor-1 levels. Increasing butyrate-producing capacity may contribute to the maintenance of blood pressure in obese pregnant women (18). The study on PROM has revealed that being infected with *Helicobacter pylori* is a risk factor for PROM (19). Furthermore, it is widely believed that infection is the primary cause of PROM, which has led to an oversight of the crucial role played by gut microbiota. The current links between gut microbiota and PROM are primarily derived from observational studies, which may be influenced by confounding factors, such as lifestyle, age, and environment (20, 21). Hence, these conditions limit the inference of causality between gut microbiota and PROM, highlighting the need for further research to elucidate their relationship.

Mendelian randomization (MR) has been widely utilized to estimate the causal association between exposure and outcome by using genetic variants as instrumental variables (IVs) (22). Genetic variants are randomly inherited from parents to offspring, making them more independent. This characteristic effectively assists MR in mitigating bias from reverse causality and confounding factors (23, 24). Large-scale genome-wide association studies (GWAS) on gut microbiota and PROM provide an opportunity for MR analysis with greatly improved statistical power.

This study aims to evaluate the potential causal association between gut microbiota and PROM using GWAS summary statistics from the FinnGen and MiBioGen consortiums through two-sample MR analysis. Our findings may identify specific pathogenic microbiota and offer new insights for early prediction and intervention in PROM.

## Materials and methods

### Study design and data sources

In the investigation, we followed the guidelines established in the STROBE-MR Statement (Guidelines for strengthening the reporting of MR studies) for reporting observational studies in epidemiology (25).

**Figure 1:**
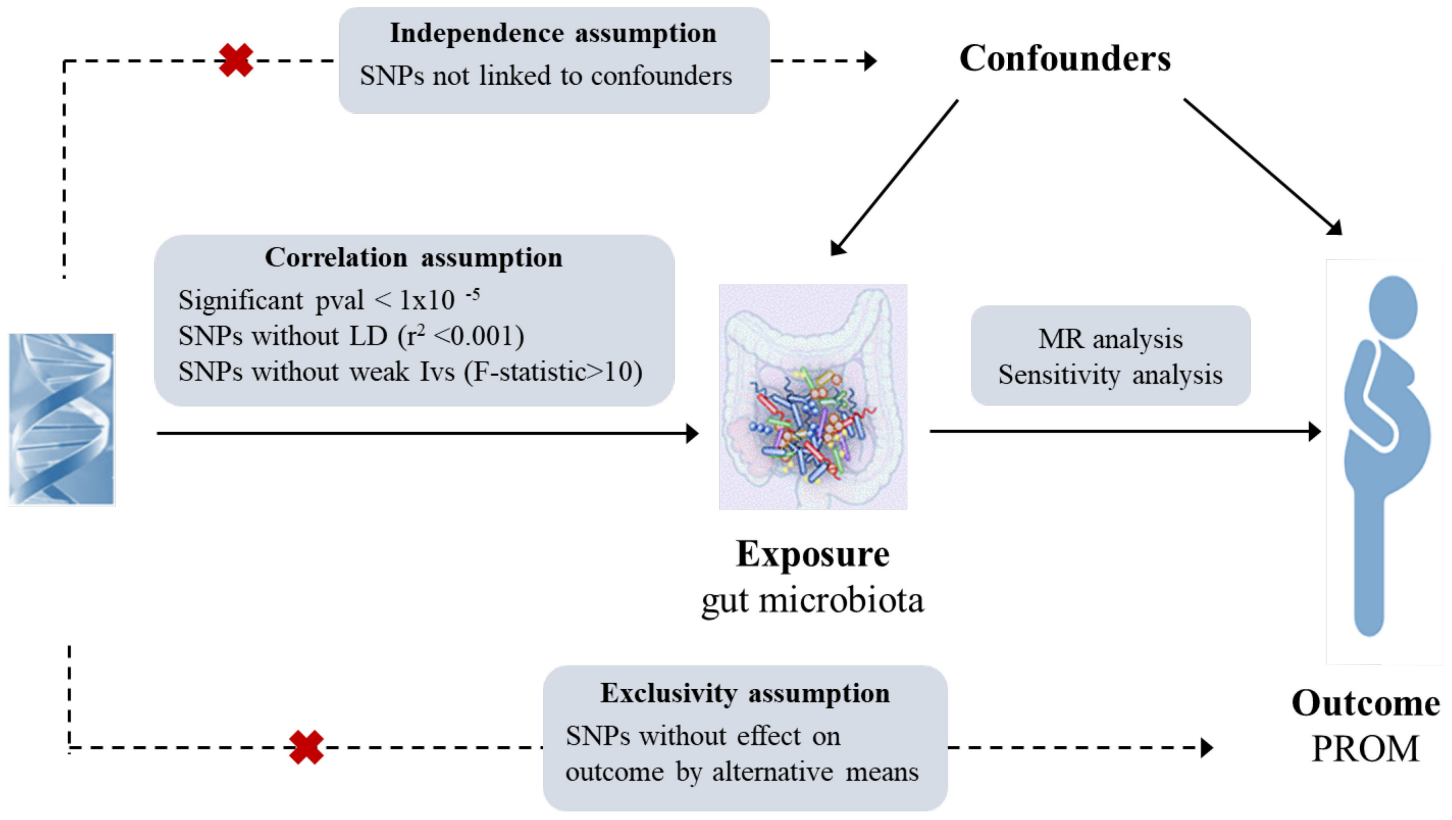
Flow chart of study design.

MR statistical analysis utilizes genetic instrumental variables (single nucleotide polymorphisms, SNPs) to infer the relationship between exposure and outcome based on three key assumptions: (1) a strong correlation between instrumental variables and exposure factors, (2) no correlation between instrumental variables and confounding factors, (3) the sole association of instrumental variables with outcomes through exposure (26, 27). The flowchart of this MR study has been shown in **Figure 1**.

Genetic data on gut microbiota was obtained from a large-scale GWAS meta-analysis conducted by the MiBioGen consortium (www.mibiogen.org), which aimed to study the influence of human genes on intestinal flora at the whole genome level and included 18340 participants from 24 cohorts, most of whom had European ancestry (Canada, Netherlands, Sweden, United States, United Kingdom, Belgium, Denmark). Bacteria contain three kinds of rRNA sequences, namely 23S, 16S and 5S. Among them, 16S rRNA is the most commonly used molecular clock in bacterial systematics because of its moderate number of nucleotides, large amount of information, high stability, and easy extraction and analysis. Variable regions (V4, V3-V4, V1-V2) of the 16S rRNA gene were used to profile the composition of gut microbiota, and microbiota quantitative trait loci (mbQTL) mapping was performed to identify the host genetic variants in the relative abundance of microbial taxa. A total of 210 taxa (9 phyla, 16 classes, 20 orders, 31 families, 119 genera, 3 unknown families, and 12 unknown genera) were extracted for analysis in this study. Further details on the gut microbiota can be found in the original study or the website (http://mibiogen.gcc.rug.nl) (28). GWAS datasets on PROM were extracted from the IEU OpenGWAS project and derived from the FinnGen consortium (http://www.finngen.fi/en), including 3011 cases and 104247 controls (19). More details (endpoint definition, mean age, and other longitudinal metrics) can be found in the FinnGen database.

All relevant data sources are publicly available. Ethical approval and participant consent were obtained in the original studies included in the GWAS, thus further ethical clearance for this study was not required.

### Instrumental variable selection

First, SNPs closely associated with gut microbiota (significant threshold *p* < 1.0×10^-5^, genetic distance = 10000 kb, r^2^ < 0.001) were screened to ensure the correlation assumption and test the effect of linkage disequilibrium (LD) and the independence of IVs (29). IVs with the F-statistics < 10 were excluded to mitigate weal instrumental bias (30). The formula of the F-statistics calculation is as follows: F= R^2^ × (n-1-k)/[(1-R^2^) × k], where R^2^ represents the portion of exposure variance explained by the IVs, k represents the number of IVs, n is the sample size. Following the above steps, the remaining SNPs were used for MR analysis (31).

### Data analysis

The data analyses were conducted by the “TwoSampleMR” package in R4.2.3. Five methods, including inverse variance weighted (IVW), MR Egger, simple mode, weighted mode, and weighted median, were employed to assess the causal association between gut microbiota and PROM. The IVW method can combine with the Wald ratio of each SNP to obtain the total effect of gut microbiota on PROM when SNP fully conforms to the three principles of MR study. Significant results obtained through the IVW method (*p* < 0.05) can be deemed credible in the absence of pleiotropy and heterogeneity, even if other methods yield non-significant findings (32). The MR-Egger method is used to evaluate potential pleiotropic effects of IVs. MR-Egger intercept analysis can better explain why this potential pleiotropy exists. If the intercept significantly deviates from zero (*p* < 0.05), it indicates the presence of horizontal pleiotropy associated with the IVs (33). This suggests that the outcome may be influenced by factors other than exposure. When the pleiotropic effect is unrelated to its genetic association with the exposure, the slope of the MR-Egger regression still offers a valid MR estimate, even in the presence of horizontal pleiotropy.

The simple mode, weighted mode, and weighted median are used as complementary methods. The simple model serves as a robust method for evaluating causal relationships between genes and phenotypes, effectively addressing potential biases. In contrast, the weighted model calculates SNP effect estimates using weights and identifies the SNP with the greatest weighted effect as the final estimate. The weighted median method takes into account the weights (inverse of standard error, SE) of IVs and calculates the median of MR-related evaluation (34).

In the sensitivity analysis, Cochran’s Q statistics with Q and p-value were used to quantify the heterogeneity in IVs, A *pval* > 0.05 indicates the absence of heterogeneity (32). Horizontal pleiotropy was evaluated using MR Egger intercept analysis, with *pval* > 0.05 indicating no pleiotropy. Outlier analysis was conducted to ascertain the presence of influential SNPs through the leave-one-out method.

For a more rigorous interpretation of causality, we employed the Bonferroni method to examine the p-value for various classifications of gut microbiotas. The results were as follows: genus p = 3.82 × 10^−4^ (0.05/131), family p =1.47× 10^−3^ (0.05/34), order p = 2.50 ×10^−3^ (0.05/20), class p = 3.13×10^−3^ (0.05/16), and phylum p = 5.56 × 10^−3^ (0.05/9).

## Results

### Instrumental variable selection

After undergoing a series of rigorous quality control procedures for IV screening, a total of 2722 independent SNPs from 210 gut microbiotas were extracted in the analysis (**Supplementary Table 1**), with statistical significance at *pval*<1.0×10^-5^, kb=10000, r2<0.001. All IVs exhibited F-statistics > 10, indicating robust IV effects and alleviating concerns of weak IV bias. Additionally, to mitigate potential confounding effects on causal inferences, PhenoScanner was utilized for screening, resulting in no exclusions of SNPs. Therefore, the genetic IVs should be deemed valid for use in this MR analysis.

### MR analysis

#### Two-sample MR analysis results

**Figure 2:**
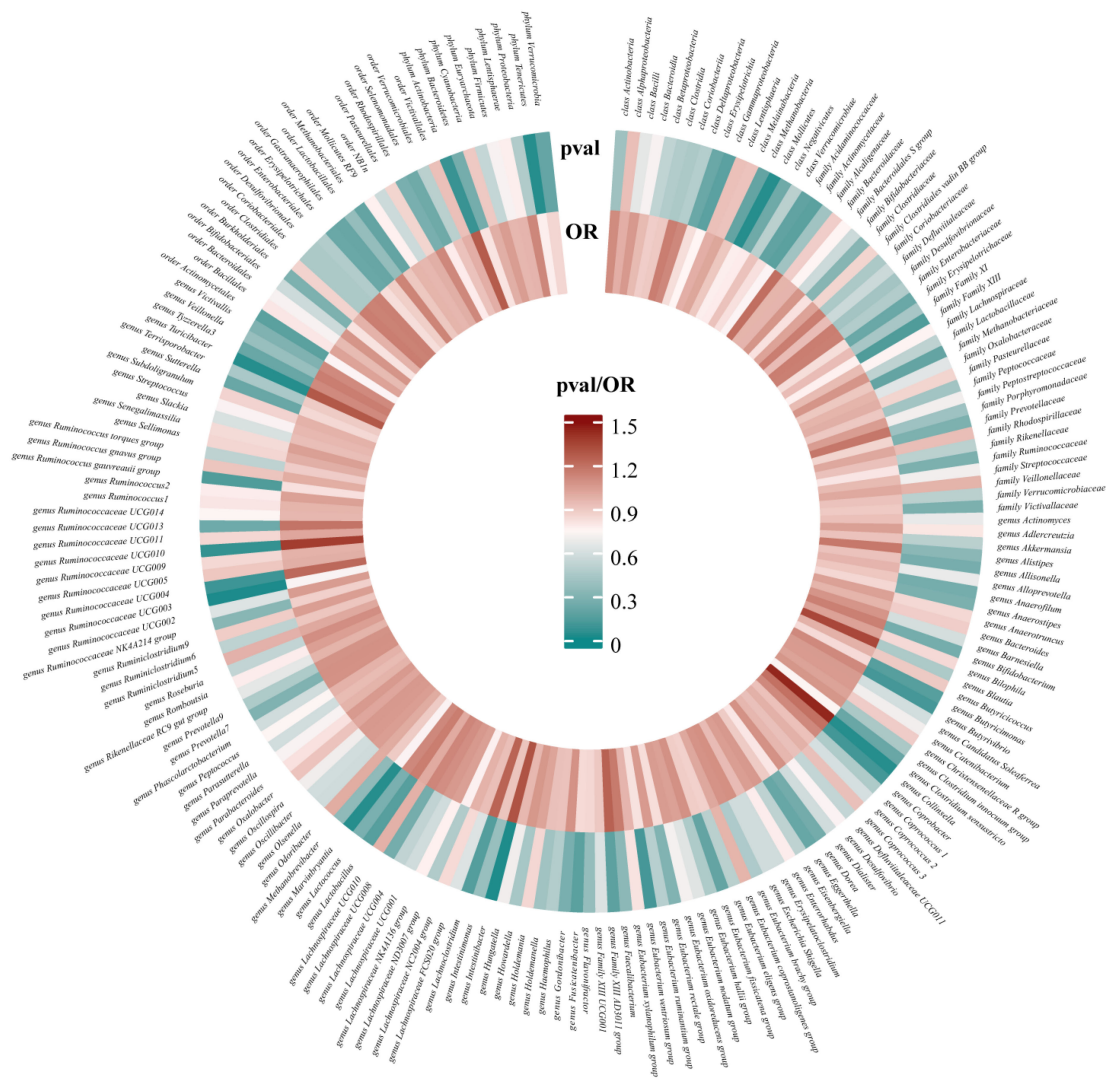
All results of IVW between gut microbiota and PROM.

**Figure 3:**
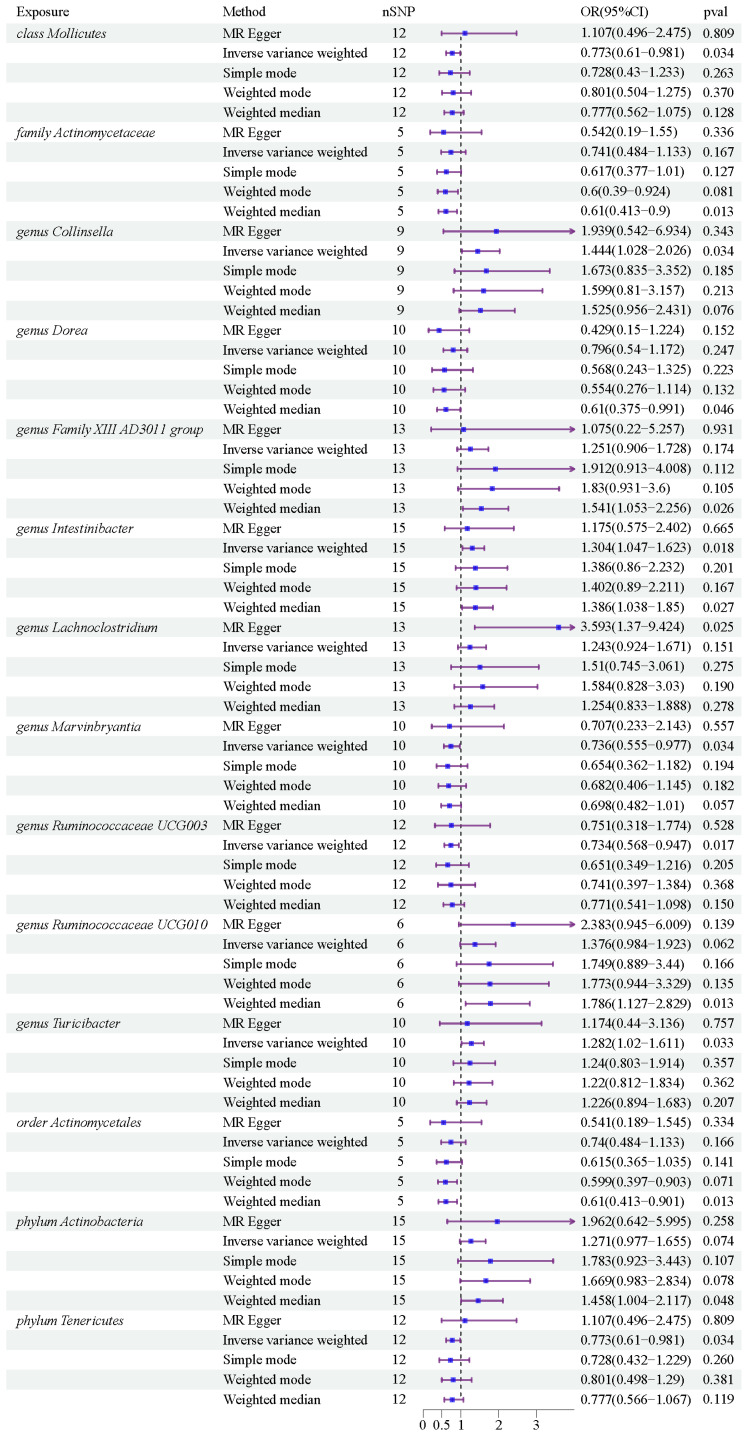
Forest plots of MR results for 14 gut microbiotas on PROM.

**Figure 4:**
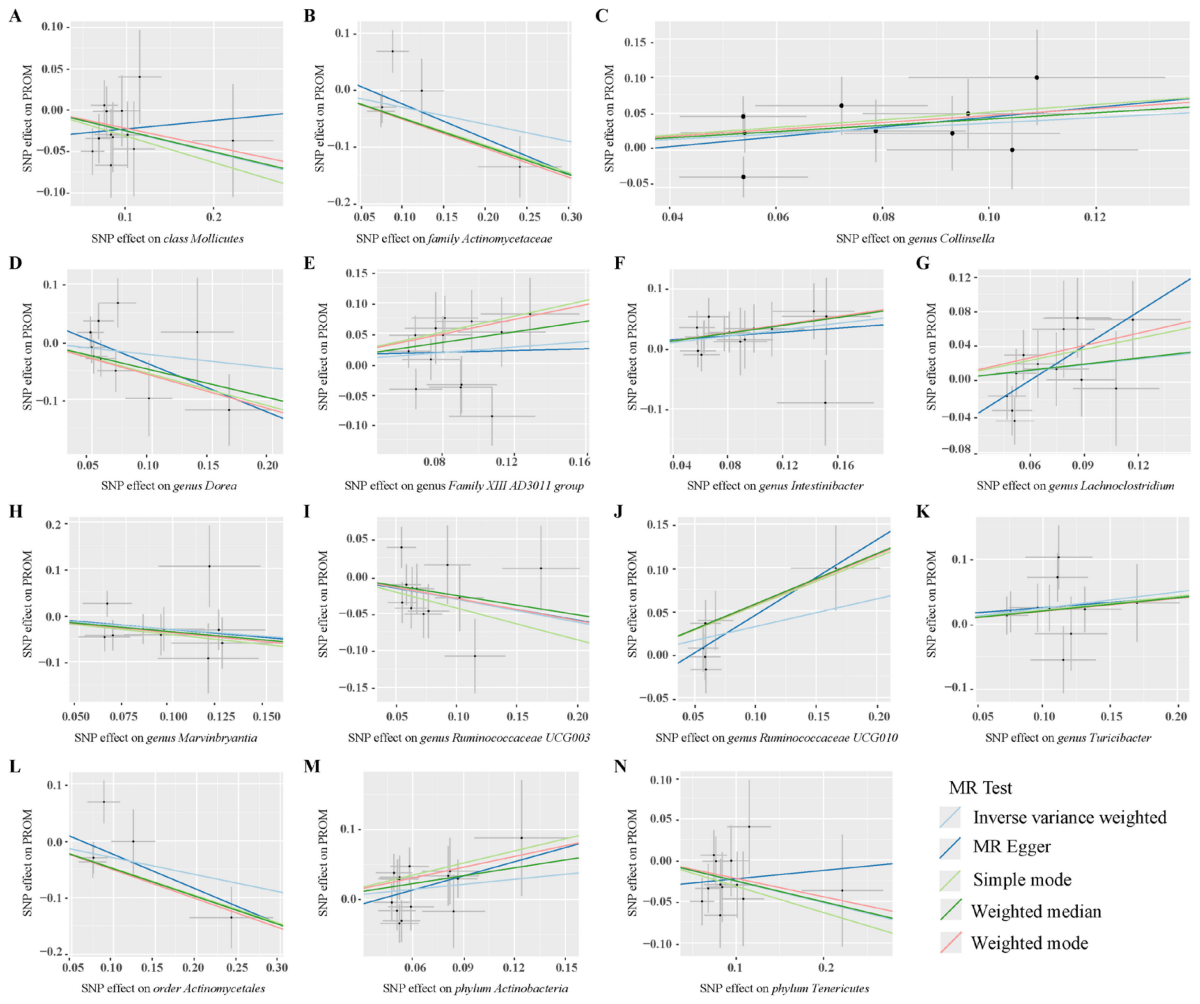
Scatter plots of MR analysis on the causal relationship between 14 gut microbiotas and PROM. **(A–N)** represents different gut microbiotas, respectively.

After conducting MR analysis, we generated a heatmap using IVW method to screen 193 gut microbiotas (**Figure 2**). This approach provides a more intuitive representation of the gut microbiota that play a significant role in PROM, as indicated by their p value and OR value. Based on the significance levels (*pval*<0.05) obtained from any of the five methods (IVW, MR Egger, simple mode, weighted mode and weighted median), a forest plot was generated, and 14 gut microbiotas (including *class Mollicutes*, *family Actinomycetaceae*, *genus Collinsella*, *genus Dorea*, *genus Family XIII AD3011 group*, *genus Intestinibacter*, *genus Lachnoclostridium*, *genus Marvinbryantia*, *genus Ruminococcaceae UCG003*, *genus Ruminococcaceae UCG010*, *genus Turicibacter*, *order Actinomycetales*, *phylum Actinobacteria*, *phylum Tenericutes*) were identified as potentially related to PROM, excluding undefined microbiotas (**Figure 3**; **Supplementary Table 2**). The scatter plots had been shown in **Figure 4**. IVW estimates suggested that the *class Mollicutes* (IVW, OR=0.773, 95%CI: 0.61-0.981, *pval* = 0.034), *genus Marvinbryantia* (OR=00.736, 95%CI: 0.555-0.977, *pval* = 0.034), *genus Ruminooccaceae UCG003* (OR=0.734, 95%CI: 0.568-0.947, *pval* = 0.017) and *phylum Tenericutes* (OR=0.773, 95%CI: 0.566-1.067, *pval* = 0.034) were associated with a reduced risk of PROM and demonstrated protective effects. Conversely, the *genus Collinsella* (OR=1.444, 95%CI: 1.028-2.026, *pval* = 0.034), *genus Intestinibacter* (OR=1.304, 95%CI: 1.047-1.623, *pval* = 0.018) and *genus Turicibacter* (OR=1.282, 95%CI: 1.02-1.611, *pval* = 0.033) were associated with an increased risk of PROM showed pathological effects.

Although the IVW method did not support the causal associations of other six gut microbiotas, Weighted median estimates revealed that several microbial taxa exhibited potential associations with PROM. Specifically, *family Actinomycetaceae* (OR=0.61, 95%CI: 0.413-0.9, *pval* = 0.013), *genus Dorea* (OR=0.61, 95%CI: 0.375-0.991, *pval* = 0.046), *genus Family XIII AD3001 group* (OR=1.541, 95%CI: 1.053-2.256, *pval*= 0.026), *genus Ruminococcaceae UCG010* (OR=1.786, 95%CI: 1.127-2.829, *pval*=0.013), *order Actinomycetales* (OR=0.61, 95%CI: 0.413-0.901, *pval*=0.013) and *phylum Actinobacteria* (OR=1.458, 95%CI: 1.004-2.117, *pval*=0.048) were found to have a suggestive association with PROM. Additionally, among these 14 gut microbiotas, MR Egger estimate of *genus Lachnoclostridium* (OR=3.593, 95%CI: 1.37-9.424, *pval*=0.02) showed a suggestive relationship with PROM as well, however, it is important to note that there was evidence of horizontal pleiotropy (*pval*=0.045).

### Sensitivity analysis

**Figure 5:**
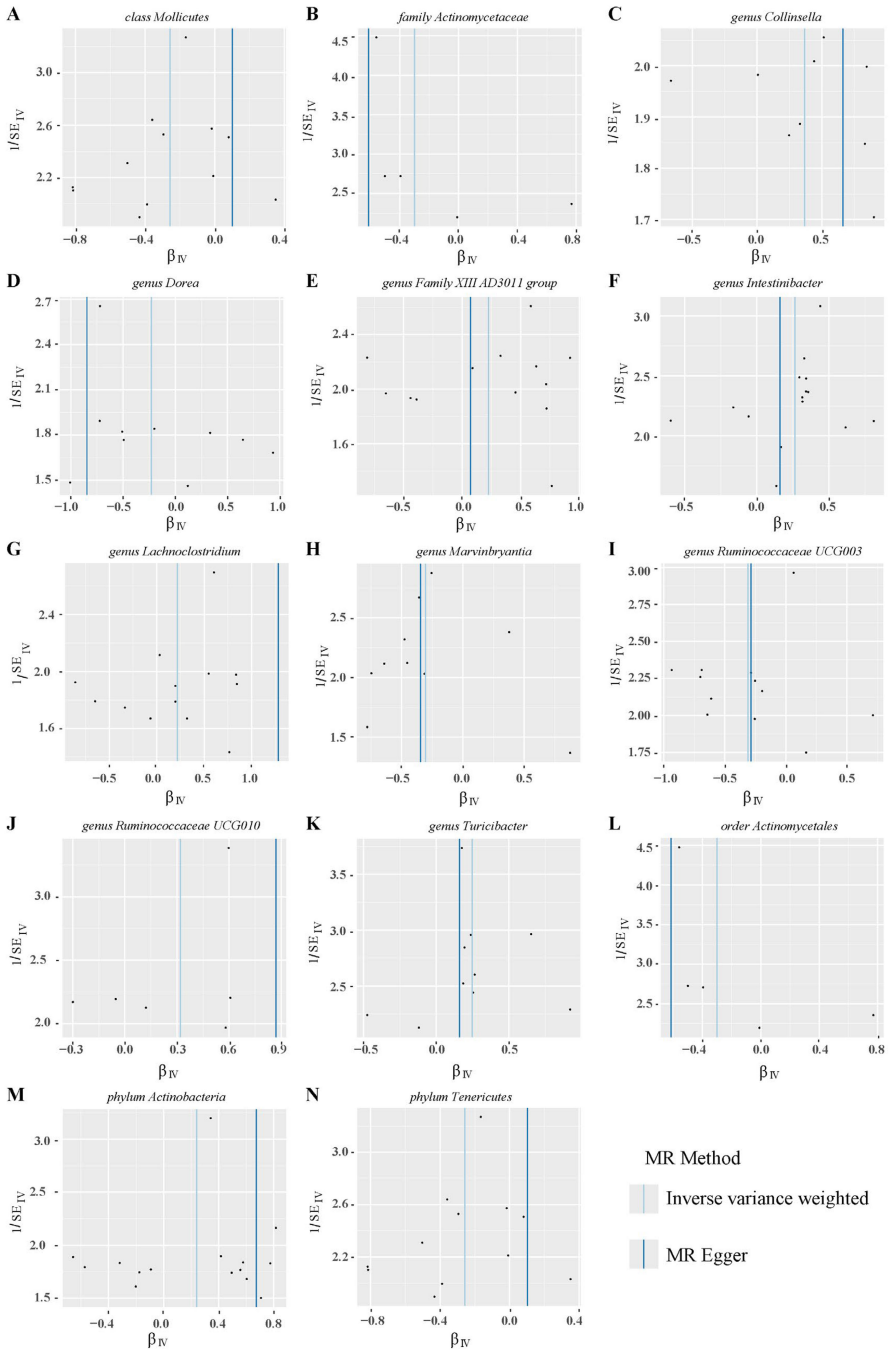
Funnel plots of heterogeneity analysis on 14 gut microbiotas and PROM. **(A–N)** represents different gut microbiotas, respectively.

Sensitivity analyses were conducted to assess the robustness of the results. IVW and MR Egger in Cochran’s Q test showed no significant heterogeneity in the IVs associated with PROM (**Figure 5** and **Supplementary Table 3**). Additionally, MR Egger intercept analysis detected horizontal pleiotropy only in *genus Lachnoclostridium* (*pval*=0.045), while no pleiotropy was found in the other 13 gut microbiotas (*pval*>0.05). The detailed results were showed in **Supplementary Table 4**.

**Figure 6:**
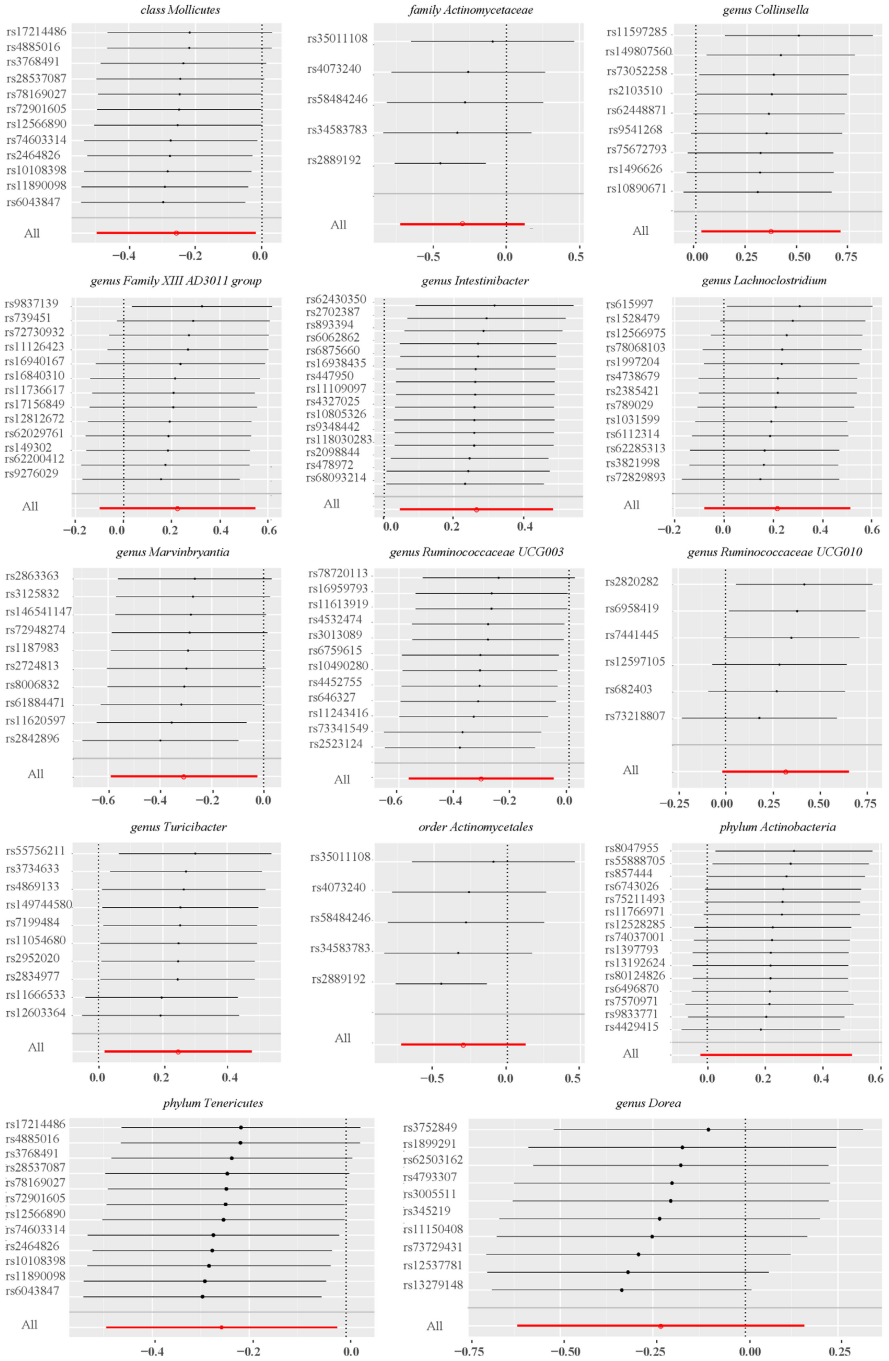
Leave-one-out plots of sensitivity analysis on 14 gut microbiotas and PROM.

Leave-one-out sensitivity analyses indicated that removing specific SNPs did not alter the causal inference outcomes, suggesting no individual IVs were solely responsible for the associations (**Figure 6**). Collectively, these findings indicated that there was no significant bias attributable to individual gut microbiota SNPs on PROM.

## Discussion

This MR study provides compelling evidence for the causal relationship between specific gut microbiota and PROM, identifying bacteria that either decrease or increase the risk. By utilizing extensive GWAS summary data from the MiBioGen consortium for gut microbiota and the FinnGen consortium for PROM, we have pinpointed specific gut bacteria that either decrease or increase the risk of PROM. These findings are consistent with existing literature, highlighting the intricate relationship between gut microbiota and pregnancy outcomes. To ensure the robustness of our findings, we employed multiple MR methods, including inverse variance weighted (IVW), MR-Egger, simple mode, weighted median, and weighted mode approaches. Sensitivity analyses such as MR-Egger intercept tests, Cochran’s Q tests, and leave-one-out analyses were performed to detect and correct for pleiotropy and heterogeneity. These methods help ensure that our results are not confounded by other factors. For example, *genus Lachnoclostridium* was excluded due to evidence of pleiotropy (*pval*=0.045), highlighting the importance of rigorous quality control in MR studies. These findings not only enhance our understanding of the link between gut microbiota and PROM, but also pave the way for new therapeutic strategies and personalized medicine in managing pregnancy complications.

Several studies have revealed the complex relationship between gut microbiota and adverse pregnancy outcomes and complications (17, 19, 35–37). This study identified 147 SNPs linked to 14 gut microbiotas associated with PROM. It was found that Mollicutes (IVW, OR=0.773, 95%CI: 0.61-0.981, *pval* = 0.034) and *Tenericutes* (IVW, OR=0.773, 95%CI: 0.566-1.067, *pval* = 0.034) were protective factors against PROM. *Mollicutes*, which include species like Mycoplasma and Ureaplasma, are known for their unique immunomodulatory properties (38–40). *Mycoplasma* and *Ureaplasma* can modulate the immune system by reducing pro-inflammatory cytokines, which may help maintain the integrity of fetal membranes and reduce PROM risk (41–44). This finding is consistent with previous studies indicating that *Mollicutes* can influence immune responses and protect against membrane rupture (45). Similarly, *Marvinbryantia* (IVW, OR=0.736, 95%CI: 0.555-0.977, *pval* = 0.034) and *Ruminococcaceae* UCG003 (IVW, OR=0.734, 95%CI: 0.568-0.947, *pval* = 0.017) also demonstrated protective effects on PROM. *Marvinbryantia* is associated with anti-inflammatory properties as it produces metabolites that have been shown to reduce inflammation (46, 47). *Ruminococcaceae UCG003* plays a crucial role in fermenting dietary fibers into short-chain fatty acids (SCFAs) like butyrate. Butyrate enhances gut barrier function and has systemic anti-inflammatory effects, which likely contribute to the strengthening of fetal membranes and reducing the risk of PROM (48, 49).

On the contrary, our study has identified specific gut microbiotas that are associated with an increased risk of PROM. *Collinsella* (IVW, OR=1.444, 95%CI: 1.028-2.026, *pval* = 0.034) was significantly associated with a higher risk. *Collinsella* has been linked to systemic inflammation and metabolic disorders, both of which can compromise gut barrier integrity (50–52). Elevated levels of *Collinsella* can disrupt gut barrier function and promote inflammatory pathways, weakening fetal membranes and increasing the risk of PROM. Additionally, *Intestinibacter* (IVW, OR=1.304, 95%CI: 1.047-1.623, *pval* = 0.018) and *Turicibacter* (IVW, OR=1.282, 95%CI: 1.02-1.611, *pval* = 0.033) were also found to be associated with an increased risk. *Intestinibacter* is associated with inflammatory conditions and has been shown to exacerbate inflammation and disrupt gut barrier function leading to weakened fetal membranes and ultimately contributing to PROM (53). *Turicibacter* has been known to influence immune responses and promote pro-inflammatory cytokines, further compromising membrane integrity thus increasing the likelihood of PROM (54, 55).

The identification of specific gut microbiota associated with PROM has significant clinical implications. These microbiotas may affect pregnancy health through various mechanisms such as metabolites, endocrine, inflammation, or immune system (41, 56, 57). The changes of gut microbiota through dietary interventions, probiotics, or prebiotics could be a viable strategy to prevent PROM. Increasing the abundance of protective bacteria such as *Marvinbryantia* and *Ruminococcaceae UCG003* through probiotic and prebiotic supplements could help maintain membrane integrity. Additionally, dietary interventions aimed at reducing harmful bacteria like *Collinsella* and *Intestinibacter* could also be beneficial in preventing PROM.

MR analysis was performed to ascertain the causal relationship between gut microbiota and PROM, effectively mitigating the influence of confounding factors. However, our study has several limitations, which could affect the interpretation of the results. Firstly, summary statistics from the public database rather than raw data were used in this MR analysis, which prevented us from performing subgroup analyses such as term PROM and preterm PROM. Secondly, the population of this study mainly focused on European ancestry, raising the possibility that the findings may not be fully applicable to other racial groups. Thirdly, due to the moderate sample size of the gut microbiota, we did not perform reverse MR analysis as it may be prone to potential instrumental biases in the findings. Fourthly, because 16S rRNA sequencing only allowed for taxonomic classification at the genus level, we were unable to investigate more specific species levels between gut microbiota and PROM. Finally, based on previous microbiota studies, we selected a relaxed p-value threshold (*pval <*1.0×10^-5^) to screen genetic instruments, which may lead to weak bias. To address this issue, we calculated the instrument strength and excluded the F statistics <10 as a conventional cutoff to mitigate potential bias effects.

Future research should focus on diverse population, detailed subgroup analysis, larger sample size, longitudinal studies tracking the gut microbiome composition during pregnancy to strengthen the important relationship between microbiotas and PROM. And the precise biological mechanisms also make us better explore the PROM’s therapeutic targets.

## Conclusion

In conclusion, this study provides strong evidence for the causal relationship between specific gut microbiota and the risk of PROM. By identifying both protective and pathogenic bacteria, our findings open new avenues for preventive strategies and therapeutic interventions, and aim at improving maternal and fetal health condition. Further research will be essential to refine these strategies and gain a comprehensive understanding of the complex interactions between gut microbiota and pregnancy.

## Data Availability

Existing datasets are available in a publicly accessible repository: Publicly available datasets were analyzed in this study. This data can be found here: (https://gwas.mrcieu.ac.uk/).
